# Reliability of prehospital patient classification in helicopter emergency medical service missions

**DOI:** 10.1186/s12873-020-00338-7

**Published:** 2020-05-25

**Authors:** A. Heino, P. Laukkanen-Nevala, L. Raatiniemi, M. Tommila, J. Nurmi, A. Olkinuora, I. Virkkunen, T. Iirola

**Affiliations:** 1 Research and Development Unit, FinnHEMS Ltd, Vantaa, Finland; 2 Department of Perioperative Services, Intensive Care Medicine and Pain Management, Turku University Hospital and University of Turku, Turku, Finland; 3 Centre for Pre-Hospital Emergency Care, Oulu University Hospital, Oulu, Finland; 4 Anaesthesia Research Group, MRC, Oulu University Hospital and University of Oulu, Oulu, Finland; 5 Emergency Medicine Services, Helsinki University Hospital, Helsinki, Finland; 6 Department of Emergency Medicine, University of Helsinki, Helsinki, Finland; 7 Emergency Medical Services, Turku University Hospital and University of Turku, Turku, Finland

**Keywords:** ASA-PS, HEMS benefit score, ICPC-2, ECOG, Prehospital, HEMS, Clinical quality registry

## Abstract

**Background:**

Several scores and codes are used in prehospital clinical quality registries but little is known of their reliability. The aim of this study is to evaluate the inter-rater reliability of the American Society of Anesthesiologists physical status (ASA-PS) classification system, HEMS benefit score (HBS), International Classification of Primary Care, second edition (ICPC-2) and Eastern Cooperative Oncology Group (ECOG) performance status in a helicopter emergency medical service (HEMS) clinical quality registry (CQR).

**Methods:**

All physicians and paramedics working in HEMS in Finland and responsible for patient registration were asked to participate in this study. The participants entered data of six written fictional missions in the national CQR. The inter-rater reliability of the ASA-PS, HBS, ICPC-2 and ECOG were evaluated using an overall agreement and free-marginal multi-rater kappa (Κ_free_).

**Results:**

All 59 Finnish HEMS physicians and paramedics were invited to participate in this study, of which 43 responded and 16 did not answer. One participant was excluded due to unfinished data entering. ASA-PS had an overall agreement of 40.2% and Κ_free_ of 0.28 in this study. HBS had an overall agreement of 44.7% and Κ_free_ of 0.39. ICPC-2 coding had an overall agreement of 51.5% and Κ_free_ of 0.47. ECOG had an overall agreement of 49.6% and Κ_free_ of 0.40.

**Conclusion:**

This study suggests a marked inter-rater unreliability in prehospital patient scoring and coding even in a relatively uniform group of practitioners working in a highly focused environment. This indicates that the scores and codes should be specifically designed or adapted for prehospital use, and the users should be provided with clear and thorough instructions on how to use them.

## Background

Clinical quality registries (CQRs) are an important part of the management and quality improvement in healthcare. Helicopter emergency medical services (HEMS) are a relatively expensive part of the healthcare system in many countries, and high-quality CQRs enable the appropriate allocation and quality improvement of HEMS units [1]. There is international consensus of the variables to be collected in HEMS datasets [2], and the systems have been collecting data on patient scoring and coding among other patient and mission related variables. Consequently, the quality control of the scoring data itself is essential as scoring systems are used to classify single patients’ clinical condition, prognosis or incident severity. The scores may be used to guide the treatment of the patient. However, the main purpose of the scoring and coding is quality control and development of the system as the patient scoring data is evaluated in larger populations.

Scoring systems used in HEMS CQRs typically include patients’ past medical history, performance status prior to the acute incident, current status, primary diagnosis and severity of the acute medical incident. In this study, we examined the following scoring and coding systems registered in the CQR in question: American Society of Anesthesiologists physical status (ASA-PS) classification system [3–5], HEMS benefit score (HBS) [6], International Classification of Primary Care second edition (ICPC-2) [7, 8] and Eastern Cooperative Oncology Group (ECOG) performance status [9, 10]. Of these, ASA-PS and ICPC-2 are used in all Scandinavian HEMS systems. ECOG is used in Finnish HEMS to describe patients physical and mental performance before acute medical incident. The prior performance status is the basis for all critical care, as it highly relates with patient ability to survive the critical care phase. HBS is used in Finland to evaluate the benefit provided by the whole prehospital system to the patient.

The aim of the current study is to evaluate the inter-rater reliability of ASA-PS, HBS, ICPC-2 and ECOG in prehospital setting.

## Methods

### Study design and participants

The data for this study was collected as all 59 physicians and paramedics working in Finnish HEMS units and responsible for patient registration were asked to anonymously fill six imaginary HEMS missions into the national CQR [11]. Study material was mailed to each HEMS base, and participants filled in the data into CQR based on this material. The entered ASA-PS, ECOG, HBS and ICPC-2 values were used for this study (Table 1*.,*supplementary material). The results on other variables have been presented in a previous study [11].

The imaginary HEMS mission scenarios were devised by authors AH, MT and TI based on clinical experience and earlier user feedback on study CQR. The cases were piloted by authors LR, AO, JN and IV and the final decision on the study cases was made by consensus. Finally, the missions included three missions with one patient and one multi-patient mission with four patients [11]. Of the four patients in multi-patient mission, most participants had filled only the most severely injured one into the CQR. This was probably attributed to the mission description: the most often registered patient was treated by a HEMS physician whereas, the other three patients were only triaged by the HEMS. Hence, the three last-mentioned patients were not taken into the analysis, and the analysis was completed with four patient descriptions on four missions. The analyzed patients represented most typical HEMS mission cases with a cardiac arrest patient, a traffic accident patient with a major trauma and a paediatric patient with seizures and an unconscious drug abuser.

### Ethics

The ethical committees of each of the five Finnish university hospital districts were contacted and verified that no ethical approval was needed for this study. All five university hospital districts gave their approval for the study. The study subjects participated voluntarily, and consent was given as they filled in the study data.

### Statistical analysis

Free-marginal multi-rater kappa (Κ_free_), was used to study the inter-rater reliability in this study setting [12–15]. Κ_free_ is an extension of the bi-rater, free-marginal kappa and uses 1/number of categories as the proportion of agreement expected by chance; Κ_free_ can take values from 1 to − 1. A value of 0 indicates a level of agreement that could have been expected by chance. Values from 0 to 1 indicate levels of agreement that are better than chance, whereas values from 0 to − 1 indicate agreement worse than chance. For calculation purposes, classes ‘not known’ and ‘missing’ were combined. In addition, an overall agreement percentage was calculated for each score and code. Analysis was done with IBM SPSS Statistics 25 and with an online Kappa calculator: http://justusrandolph.net/kappa/.

## Results

All 59 Finnish HEMS physicians and paramedics responsible for patient registration were invited to participate in this study, of which 43 responded and 16 did not answer. One participant was excluded due to unfinished data entering. We analysed all patient scoring and coding data of the included 42 participants, but one participant had not registered the the multi-patient mission patient chosen for the analysis, thus resulting in missing data for one patient.

ASA-PS resulted in an overall agreement of 40.2% and Κ_free_ of 0.28 [95% CI 0.12, 0.44] (Table 1.). Most ASA-PS variation was in the case of an unconscious drug abuser: 15 participants scored the patient ASA-PS I or II, but some participants also scored the patient as ASA-PS III or IV.

**Table 1:** ASA-PS distribution of four patients as recorded by 42 physicians and paramedics in HEMS database

***Patient type***	ASA I	ASA II	ASA III	ASA IV	ASA V	ASA not known	ASA missing
Cardiac arrest	4	24	6	0	1	7	
Major trauma	32	5	0	0	1	3	1
Paediatric seizures	6	21	14	0	0	1	
Drug abuse, unconscious	15	15	4	1	0	7	

*ASA I* “A normal healthy patient”, *ASA II* “A patient with a mild systemic disease”, *ASA III* “A patient with a severe systemic disease”, *ASA IV* “A patient with a severe systemic disease that is a constant threat to life”, *ASA V* “A moribund patient who is not expected to survive without the operation”

HBS had an overall agreement of 44.7% and Κ_free_ of 0.39 [95% CI 0.26, 0.51] (Table 2.). Most variations were observed in the paediatric patient, with study participants scoring HBSs from HBS 3 to HBS 8.

**Table 2:** HBS distribution of four patients as recorded by 42 physicians and paramedics in HEMS database

***Patient type***	HBS 3	HBS 4	HBS 5	HBS 6	HBS 7	HBS 8	HBS missing
Cardiac arrest	0	0	1	6	30	5	0
Major trauma	3	2	0	18	5	13	1
Paediatric seizures	3	26	0	13	0	0	0
Drug abuse, unconscious	1	1	0	13	27	0	0

ICPC-2 coding had an overall agreement of 51.5% and Κ_free_ of 0.47 [95% CI 0.28, 0.67] (Table 3.). The cardiac arrest patient had the most variations in the ICPC-2 as the participants registered five different codes for this patient.

**Table 3:** ICPC-2 distribution of four patients as recorded by 42 physicians and paramedics in HEMS database

***Patient type***	N88 Epilepsy	P19 Drug abuse	N07 Convulsion/ Seizure	A96 Death	A84 Poisoning by medical agent	A81 Multiple trauma/ injuries	D80 Injury digestive system other	K80 Cardiac arrhythmia NOS	K99 Cardiovascular disease other	N79 Concussion	A80 Trauma/Injury NOS	A10 Bleeding/haemorrhage NOS	ICPC2 missing
Cardiac arrest	0	0	0	**3**^**c**^	0	0	0	**14**	**23**	**1**	**1**^**f**^	0	0
Major trauma	0	0	0	0	0	**23**^**d**^	**1**	0	0	0	**12**	**5**^**g**^	**1**
Paediatric seizures	**5**	0	**37**^**b**^	0	0	0	0	0	0	0	0	0	0
Drug abuse, unconscious	0	**25**^**a**^	0	0	**16**	0	0	0	0	**1**^**e**^	0	0	0

Study CQR enables an additional ICPC-2 with the primary code:

^a^two participants additionally coded A84

^b^two participants additionally coded N88

^c^one participant additionally coded K99

^d^one participant additionally coded N80 (head injury other) and one participant additionally coded A10

^e^one participant additionally coded P19

^f^one participant additionally coded A88 (adverse effect physical factor)

^g^one participant additionally coded A80

ECOG had an overall agreement of 49.6% and Κ_free_ of 0.40 [95% CI 0.11, 0.68] (Table 4.). Similar with HBS, ECOG also had the most variations with the paediatric patient. The participants registered this patient from ECOG grades 0 to 4, and eight participants registered the ECOG for this patient as not known.

**Table 4:** ECOG distribution of four patients as recorded by 42 physicians and paramedics in HEMS database

***Patient type***	Grade 0	Grade 1	Grade 2	Grade 3	Grade 4	Not known	Missing
Cardiac arrest	22	5	1	0	0	14	
Major trauma	38	0	0	0	0	3	1
Paediatric seizures	18	9	4	2	1	8	
Drug abuser, unconscious	29	5	0	0	0	8	

0 Fully active, able to carry on all pre-disease performance without restriction

1 Restricted in physically strenuous activity but ambulatory and able to carry out work of a light or sedentary nature, e.g., light house work, office work

2 Ambulatory and capable of all selfcare but unable to carry out any work activities; up and about more than 50% of waking hours

3 Capable of only limited selfcare; confined to bed or chair more than 50% of waking hours

4 Completely disabled; cannot carry on any selfcare; totally confined to bed or chair

### Discussion

The aim of the current study was to evaluate the inter-rater reliability of the ASA-PS, HBS, ICPC-2 and ECOG in a prehospital setting. The results demonstrate that the prehospital ICPC-2 has moderate, and the ASA-PS, HBS and ECOG poor, inter-rater agreement amongst HEMS physicians and paramedics.

The results are not unexpected, as no complete patient medical history is available, and time to gather information in a prehospital setting is limited, especially in critical situations. In addition, the ASA-PS, ICPC-2 and ECOG were not originally built for use with prehospital patients. Nonetheless, these scores are constantly used for scientific and quality control purposes also in prehospital settings.

In this study, the ASA-PS and ECOG demonstrated very low inter-rater reliability. Many participants registered the ASA-PS or ECOG as ‘not known’. Imitating real-life prehospital work, the lack of patients’ medical history while registering, could explain the relatively high number of participants unable to assess the ASA-PS and ECOG. It is also possible that participants’ personal opinions of these scores may have influenced their willingness to register them. Moreover, the time of assessment may not have been clear to participants: some may have scored based on the patients’ past medical history and others on the patients’ acute status. This variation, however, could be corrected with more detailed instructions and training. Regardless of the reason for the poor results, the reliability of the ASA-PS and ECOG is questioned, and their value in prehospital use should certainly be reconsidered.

The HBS indicated poor inter-rater reliability in this study, in contrast to a previous study that demonstrated markedly higher inter-rater reliability [6]. Of note, in contrast to the earlier study which included more routine patient cases, the cases in this study were intentionally more problematic, as the study was designed to reveal possible weaknesses of the studied CQR. Nonetheless, the inter-rater reliability was below all our expectations, indicating that the HBS needs to be updated or re-implemented thoroughly. Indeed, the original definitions containing patient case examples are nearly 20 years old and are no longer valid, as prehospital care has significantly changed and evolved over time (Table 2., supplementary material).

The ICPC-2 has already been implemented in many EMS systems, and it is a variable that is recommended to be collected in all Scandinavian EMS systems [16]. Based on the moderate inter-rater agreement found in this study, it can be argued that it is not reasonable to use the ICPC-2 in prehospital care in its existing form. Indeed, the ICPC-2 has been adjusted for prehospital use by the Nordic expert group [16], but, to the best of our knowledge, it has not been published yet.

The ASA-PS, ICPC-2 and ECOG are used to classify prehospital patients, and the HBS is used to evaluate the benefit of prehospital care. The questions raised by our results do not mean that prehospital patient scoring should be discontinued, but more detailed instructions and more intense staff training and data quality monitoring are clearly needed. Prehospital access to electronic patient records can facilitate and improve patient scoring and coding. Indeed, this will be a reality in Finland in the next few years. Most importantly, the scores used should be designed or adapted for prehospital usage.

## Limitations

The main limitation of this study is that the scenarios were fictional and simulated in a written form. This can never equal real-life patient contact on an actual HEMS mission. However, the material was given in a form that equates to real-life documentation in the Finnish prehospital system, and data were collected with a system that is identical to a real-life CQR.

## Conclusions

This study showed poor inter-rater reliability in prehospital patient scoring and coding by a relatively uniform group of practitioners working in a highly focused environment. This indicates that the scores and codes should be specifically designed or adapted for prehospital use, and the users should be provided with clear and thorough instructions on how to use them.

## Supplementary information

**Additional file 1.**

## Data Availability

The datasets generated and analysed during the current study are available from the corresponding author on a reasonable request.

## Abbreviations

ASA: American Society of Anesthesiologists
ASA-PS: American Society of Anesthesiologists physical status
CQR: Clinical quality registry
ECOG: Eastern Cooperative Oncology Group
EMS: Emergency medical service
HEMS: Helicopter emergency medical service
ICD: International Statistical Classification of Diseases and Related Health Problems
ICPC: International Classification of Health Process in Primary Care
WONCA: World Organization of National Colleges, Academies and Academic Associations of General Practitioners/Family Physicians
