# Supplementary material for: Reliability of prehospital patient classification in helicopter emergency medical service missions
Source: BMC Emerg Med. 2020 May 25;20:42. doi: 10.1186/s12873-020-00338-7 (PMC7249641; doi:10.1186/s12873-020-00338-7)
Supplement: Supplementary file 1 — Additional file 1. [file 12873_2020_338_MOESM1_ESM.pdf]

Table 1. Patient scores and codes in the HEMS CQR

	Original application	Original introduction	Number of categories
ASA-PS	Assessment of patients physical perioperative fitness	1941	6
HEMS Benefit Score	Evaluate the benefit of EMS system to a patient	1997	9
ICPC-2	Reason for encounter, diagnose and process of care in primary health care	1987	17 chapters divided in 7 components
ECOG	Performance status scale for oncology patients	1960	5

Table 2. HEMS Benefit Score

0	The patient was not seen
1	Prehospital care was not deemed necessary
2	Prehospital care apparently had no significance from the patient's standpoint (e.g., cannulation, no medication or fluid therapy) or despite prehospital care the patient died before reaching the hospital
3	Prehospital care apparently had no significance from the standpoint of the prognosis, but the patient's symptoms or pain was alleviated (e.g., injured patient's analgesia)
4	Prehospital care was administered; its significance from the patient's standpoint is unknown, difficult to assess or only assessable retrospectively (e.g., treatment of ischaemic chest pain, brief convulsions, mild breathing difficulty)
5	Without prehospital care (administered by the first response unit or the physician staffed unit), the patient would have died before reaching the hospital, but he/she is assessed as having a poor prognosis (e.g., serious brain damage, coma caused by spontaneous cerebral haemorrhage, primary survival from cardiac arrest after lengthy response times, terminal phase of a malignant disease)
6	The patient was given prehospital care that can be assessed to reduce mortality or otherwise improve the prognosis (see appendix)
7	Without prehospital care (administered by the first response unit or the physician staffed unit), the patient would have died before reaching the hospital, and he/she cannot be assessed as having a poor prognosis (see appendix)

8	Category 7 in situations where other emergency medical staff on site would not have been capable of administering the aforementioned life-saving treatment

NOTE

- prehospital care = speed and/or quality of treatment and/or transport
- the basis must be assessment of benefit to the patient, not the demandingness or duration of the treatment
- the assessment must be done immediately after the operation, using available information, and the assessment must not be changed on the basis of information received later

Table 3. HEMS benefit score, application guideline

Disease / injury	Score
Uncomplicated ST elevation myocardial infarction	
• Prehospital GP inhibitor, heparinoid and PCI < 90 min from emergency call	6
• Thrombolysis 0–4 h from onset of pain	6
• Thrombolysis > 4 h from onset of pain	4
Complicated ST elevation myocardial infarction	
• Whenever prehospital care has a positive response in haemodynamics	6–8
Cardiac arrest	
• No ALS attempted	1
• ALS attempted but the patient died	2
• Primarily survived normothermic adult	
o VF ongoing when the physician encounters the patient	7–8
o Found with asystole / PEA regardless of delays	5
o BLS > 10 min or ALS > 20 min or ROSC > 30 min	5
o BLS < 10 min and ALS < 20 min and ROSC < 30 min	7–8
Breathing difficulty without an injury	
• Pulmonary oedema and aggravated COPD	
o SpO_2 < 80 % on encounter and treatment has a positive response	
o Decreasing SpO_2 regardless of administered oxygen	6–8
• Status asthmaticus	6–8
o SpO_2 < 90 % on encounter and prehospital care has a positive response	
o Decreasing SpO_2 regardless of administered oxygen	
• Other aetiology; no previous incurable disease	
o SpO_2 < 85 % on encounter and treatment has a positive response	6–8

	6–8<br>6–8
Injury<br>• Treatment of hypovolaemia > 1500 ml or > 20 ml / kg<br>• Securing the airway by intubation<br>• Capnography controlled ventilation as treatment of elevated ICP<br>• Successful drainage of the pleural cavity because of desaturation<br>• Successful drainage of tension pneumothorax<br>• Time savings > 30 min with helicopter transport of a multiple-injured patient<br>• Crucial time savings > 10 min with helicopter transport in a case of critical hypovolaemia caused by a penetrating injury (-> emergency surgery)	6–8<br>6–8<br>6<br>6–8<br>6–8<br>6–8<br>8
Status epilepticus and hypoglycaemia<br>• Duration > 30 min; the patient awoke after glucose infusion<br>• Status epilepticus; treated with general anaesthesia<br><br>Unconsciousness without injury<br>• Suspected intracranial haemorrhage and coma (GCS 3–5 /15)<br>• Securing the airway by intubation and controlled ventilation, if there is no reason to consider the patient's status as having a poor prognosis	6<br>6<br><br>5<br>6

GP inhibitor, Glycoprotein IIb/IIIa Inhibitor; PCI, Percutaneous Coronary Intervention; ALS, Advanced Life Support; VF, Ventricular Fibrillation; PEA, Pulseless Electrical Activity; BLS, Basic Life Support; ROSC, Return of Spontaneous Circulation; COPD, Chronic Obstructive Pulmonary Disease; SpO_2, Oxygen saturation measured by pulse oximetry; ICP, Intracranial Pressure; GCS, Glasgow Coma Score.
